# Path Diversity Improved Opportunistic Routing for Underwater Sensor Networks

**DOI:** 10.3390/s18041293

**Published:** 2018-04-23

**Authors:** Weigang Bai, Haiyan Wang, Ke He, Ruiqin Zhao

**Affiliations:** 1 Key Laboratory of Ocean Acoustics and Sensing, Northwestern Polytechnical University, Ministry of Industry and Information Technology, Xi’an 710072, China; hk@nwpu.edu.cn (K.H.); rqzhao@nwpu.edu.cn (R.Z.); 2 School of Marine Science and Technology, Northwestern Polytechnical University; Xi’an 710072, China

**Keywords:** underwater sensor networks, opportunistic routing, candidates selection, candidate coordination

## Abstract

The packets carried along a pre-defined route in underwater sensor networks are very vulnerble. Node mobility or intermittent channel availability easily leads to unreachable routing. Opportunistic routing has been proven to be a promising paradigm to design routing protocols for underwater sensor networks. It takes advantage of the broadcast nature of the wireless medium to combat packet losses and selects potential paths on the fly. Finding an appropriate forwarding candidate set is a key issue in opportunistic routing. Many existing solutions ignore the impact of candidates location distribution on packet forwarding. In this paper, a path diversity improved candidate selection strategy is applied in opportunistic routing to improve packet forwarding efficiency. It not only maximizes the packet forwarding advancements but also takes the candidate’s location distribution into account. Based on this strategy, we propose two effective routing protocols: position improved candidates selection (PICS) and position random candidates selection (PRCS). PICS employs two-hop neighbor information to make routing decisions. PRCS only uses one-hop neighbor information. Simulation results show that both PICS and PRCS can significantly improve network performance when compared with the previous solutions, in terms of packet delivery ratio, average energy consumption and end-to-end delay.

## 1. Introduction

Underwater sensor networks (UWSNs) have attracted strong attention from both researchers and industry in fields such as oceanographic data collection, pollution monitoring, offshore exploration, disaster prevention, assisted navigation, tactical surveillance, marine fish farms, etc. [1,2,3,4]. UWSNs are a new research paradigm that poses exciting challenges compared to the ground-based existing networks due to the intrinsic properties of underwater environments [5]. The pre-defined routing protocols cannot be applied to UWSNs successfully because the characteristics of the underwater acoustic physical channel, including limited bandwidth, high propagation delay, high bit error rate (BER) and temporary loss of connectivity, may result in high energy consumption and weak reliability. When packets are transmitted along the pre-defined route, once a partial communication link is shut down, the entire path will be unreachable.

**Figure 1:**
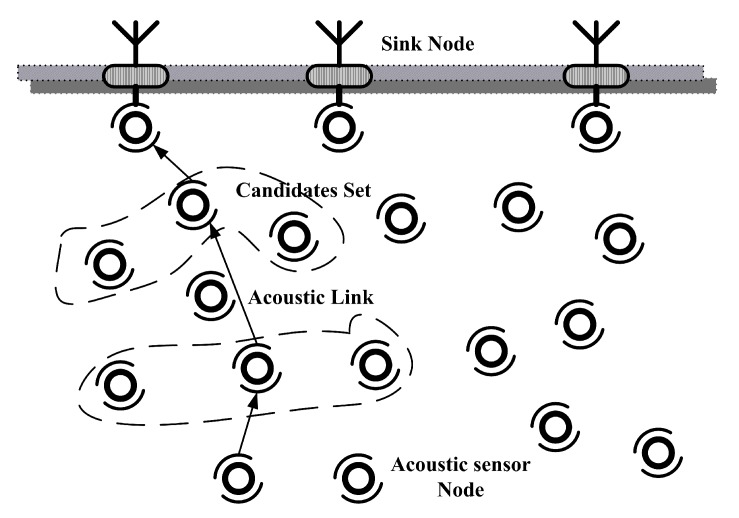
Opportunistic routing diagram.

Comparing traditional routing protocols, opportunistic routing (OR) has been proven as a promising paradigm to design routing protocols for UWSNs [6]. The main idea of OR is shown in Figure 1. When a sensor has packets to deliver, instead of a unique next-hop forwarder in traditional multi-hop routing, OR selects a set of next-hop candidate nodes on the fly at each hop to forward packets. OR can combat packet losses by taking advantage of simultaneous packet reception among the candidate set. Then, the packets will be forwarded in a prioritized way towards the destination [7].

Candidate set selection plays a significant role in OR protocols. It handles selecting a subset of neighboring nodes to continue to forward the packet toward the destination [6]. Candidate selection methods in existing works can be divided into three categories: geographic-based [8,9,10,11,12], depth-based [9,13] and EPA-based [14,15,16]. Geographic-based methods take advantage of node’s location to forward packets. It can limit the forwarding direction and the forwarding range to save energy (e.g., VBF [9] and HH-VBF [10]). They use the ideal assumption that the geographical information of nodes can be obtained easily. Actually, finding the location information of nodes is the primary challenge as global positioning system (GPS) waves in the 1.5 GHz band do not propagate through water. Further, fine-grained localization by underwater positioning techniques with limited energy expenditure is difficult [17]. Depth-based methods always take the depth information to make routing decision locally. It does not require topology or route information. Without effective retransmission suppression, the depth-based selection method applied in DBR protocol [13] results in too much retransmission. That is not suitable for UWSNs due to the higher energy consumption. The EPA-based methods employ the forwarding progress and underwater fading channel to chose forwarding nodes. The EPA (Expected Packet Advance) metric [18] is defined as packet forwarding advancements provided by the neighboring nodes. In Hydrocast [14], a heuristic algorithm is proposed to find a neighboring set with the maximum normalized sum of advancements to forward packets. Although the EPA-based method can provide good solutions to against the underwater unreliable communication channel, a major issue is ignoring the effect of candidates location distribution and candidate set size on path reliability.

The routing void problem poses a challenge to OR protocols. It occurs whenever the current forwarders have no neighbor node closer to the destination than itself. There are various reasons for the presence of void areas, such as sparse topology, temporary obstacles, unreliable nodes or links, etc. [16]. Many void-handling techniques for routing protocols have been proposed in UWSNs [19]. Several techniques, such as flooding techniques, backward forwarding, transmission power adjustment, network topology control, etc., are proposed to recover path when the forwarder located at the local maximum point. Generally, OR protocols can provide multiple potential candidates for packet forwarding. Void nodes can take themselves out of the packet forwarding to provide the opportunity for other available nodes [20], which is called passive participation void-handling technique [19]. Thus, a well-designed candidates location is more important to improve the path reliability.

In this paper, we propose a path diversity improved strategy in candidates selection procedures. This strategy takes the impact of channel quality on packet reception and the influence of candidates location distribution on path connectivity into account. Our main goal is finding an available candidate set with the maximum advancements and diverse forwarding path towards the next-hop to improve packet forwarding efficiency. Then, we propose two effective location-free routing protocols, position improved candidates selection (PICS) and position random candidates selection (PRCS), for UWSNs based on the path diversity improved strategy. PICS and PRCS adopt different candidate selection range, which leads to different candidate set selection algorithm and candidate coordination mechanism. Both PICS and PRCS employ the sender-based scheme in candidate set selection. The current forwarder selects a candidate set at each hop with the information provided by the periodic beacon and broadcasts the packets to them. The routing information, including candidate members and coordination scheme, is embedded in the packet header. When the neighbor nodes receive the packets, they first check the header of the packet. If they belong to the current candidate set, they will forward the packets based on the coordination scheme. Otherwise, they remain silent. This process is repeated until the packets arrive at the sink nodes. Finally, we show the path diversity improved strategy can efficiently improve routing performance by simulation: PICS and PRCS outperform previous solutions, i.e., DBR, EPA (Hydrocast) and OVAR, in terms of packet delivery ratio, energy efficiency and end-to-end delay.

The rest of the paper is organized as follows. In Section 2, we briefly review some related works. We introduce the path diversity improved opportunistic routing in detail in Section 3, including path diversity improved forwarding mechanism and the candidate coordination methods. The simulation results are shown in Section 4. In Section 5, we conclude this paper.

## 2. Related Work

Generally, routing protocols for UWSNs are mainly classified into three types including geographic-based, depth-based and link-state based routing protocols. Here, we consider two important factors: candidates selection procedures and candidate coordination to survey the routing protocols.

Geographic-Based Routing Protocol-Vector-based forwarding (VBF) routing protocol [9] assumes every node already knows its location. It employs the geographic-based candidate selection strategy, and the nodes are allowed to forward packets when its distance to the forwarding vector is less than a pre-defined radius, where the forwarding vector is guided by the location from the source to the destination. A TDMA-based candidate coordination is applied, and the holding timer depends on the distance to the forwarding vector. An enhanced version, HH-VBF [10], is proposed for sparse network scenarios. It uses a dynamic pipeline at each hop originated from every intermediate node to the destination. VBVA is also a vector-based routing protocol, which is proposed to address the void problem in mobile network scenarios. It uses vector-shift and back-pressure mechanisms to bypass a concave void. HH-VBF and VBVA employ the same candidate selection scheme with VBF, but adopt different forwarding vector. The focused bream routing (FBR) protocol [8] assumes every node has its location information and every source node knows the final destination location. Candidate nodes are those that lie within a cone of angle emanating from the transmitter towards the final destination. The RTS-CTS based candidate coordination solution is applied. In another geographic-based routing protocol, introduced in [12], a greedy forwarding strategy, neighbors that have the smallest distance to the sonobuoy will be selected as the next-hop forwarder. The centralized and distributed topology control methods by adjusting the depth of nodes are proposed to reduce the impact of the routing void problem and improve packet forwarding efficiency.

Depth-Based Routing Protocol-Depth-based routing (DBR) protocol [13] employs depth-based candidate selection strategy. It is a greedy forwarding strategy where nodes are allowed to forward packets when their depth is lower than that embedded in the packets. Otherwise, they discard the packets. A TDMA-based candidate coordination is applied, and the holding timer depends on the depth between the forwarder and itself. HydroCast [14] uses the EPA-based candidate selection metric, which is the normalized sum of advancements made by this neighboring set. The normalized advancement is associated with the packet delivery probability and the progress to the destination. The candidate selection algorithms proposed in [14] try to find a forwarding set of hidden-terminal free nodes that maximizes EPA. It adopts normalized advance (NADV) metric [21] as the priority metric. The linear holding timer for candidate coordination depends on the depth of the receiver. In HydroCast, a routing recovery technique is proposed to deal with the maximum voids problem. The local maximum node will search for a path to a lower depth node using 2D surface flooding. Void-aware pressure routing (VAPR) protocol [15] employs an enhanced beacon technique to build a directional trail to the closest sonobuoy. It uses the sequence number, hop count and depth information in periodic beacons to set up next-hop direction. When a beacon is received from a shallower depth node, the direction is set as up. Otherwise, it is set as down. The candidate selection algorithm tries to find the cluster with the highest expected packet advancement toward the selected direction. Opportunistic void avoidance routing (OVAR) protocol [16] takes advantage of the distributed beacon to construct the adjacency graph at each hop. The sink node initiates the beacon process, and the receiving node decides how to deal with the beacon according to hop count value of the beacon and itself. Gradually, the beacon will be cascaded down to the network. OVAR aims to extract a forwarding set with maximum EPA from the adjacency graph, which excludes any hidden node. Both VAPR and OVAR adopt EPA-based candidate selection strategies similar to HydroCast, the main difference being the local neighbor information.

Link-State Routing Protocol-A location unaware message forwarding technique [17] uses independent and local forwarding decisions to select next hop forwarder on-the-fly based on its link transmission reliability and reachability to the gateway, which is estimated by exchanging one-hop control packets. Moreover, an accumulate-and-forward concept is applied to deal with the large propagation delay. An enhanced version of the work in [17] proposed in [22] adopts an adaptive technique to reduce the end-to-end delay by dynamically calculating the data hold time at each node. The metrics include current buffer occupancy, the delay experienced by stored data packets, arrival and service rate, data transmissions from neighbors and reachability. A location-free link state routing (LLSR) [23] uses a greedy hop-by-hop routing by relying on the parameters such as hop count, path quality, and pressure, which is supplied by the periodical beacon. In different transmission cases, the three parameters will be used respectively to select the next-hop forwarder.

As mentioned before, the geographic-based routing protocols require the underwater positioning techniques. Because of the time-varying characteristics of underwater acoustic channels and the higher propagation delay of the acoustic signals, the link-state routing protocols should be supported by the practical link-state estimation techniques. In this paper, instead of geographic information, each node can provide its depth information by the pressure sensor, similar to the depth-based routing protocols. Different from the previous works on the candidate set selection, we focus on the effect of candidates location distribution and candidate set size on path reliability.

## 3. Path Diversity Improved Opportunistic Routing

### 3.1. System Model

We model a typical 3D underwater sensor network composed of some autonomous underwater sensor nodes and sink nodes, where sensor nodes transmit sensed information to the sink nodes on the surface through the wireless acoustic communication channel. Assume that the nodes are enumerated as N={1,2,…,N}, the depth of node *i* is denoted as $d_{i}$, and the Euclidean distance between node *i* and node *j* is represented by $d_{ij}^{e}$. The neighbor set of node *i* is denoted as $V_{i}$ and the candidate set of node *i* is denoted as $C_{i}$. The node communication range is *R*. $\tau_{ij}=d_{ij}^{e}/c$ is propagation delay between node *i* and node *j*, while *c* is the acoustic speed in water.

We consider a lossy channel in which path loss and bit error depend on the traversed distance and signal frequency [24]. The path loss over a distance *r* for a signal of frequency *f* due to large scale fading is given as:

(1) $$A\left(r,f\right)=n\cdot 10\log r+\alpha \left(f\right)r\times 10^{-3}$$

The first term in the above summation represents the spreading loss, and the second term represents the absorption loss. The spreading factor *n* describes the geometry of propagation, and its commonly used values are n=2 for spherical spreading, n=1 for cylindrical spreading, and n=1.5 for the so-called practical spreading. The absorption coefficient α(f) can be expressed empirically, using the Thorp’s formula, which gives α(f) in dB/km for *f* in kHz.

(2) $$\alpha \left(f\right)=\frac{0.11f^{2}}{1+f^{2}}+\frac{44f^{2}}{4100+f^{2}}+3.0\times 10^{-4}f^{2}+3.3\times 10^{-3}$$

The average signal-to-noise ratio (SNR) over distance $d_{si}^{e}$ is given by:

(3) $$\gamma \left(s,i\right)=\frac{PA(d_{si}^{e},f)}{N(f)\Delta f}$$

where *P* is the transmitter power, N(f) is the ambient noise and Δf represents the receiver noise bandwidth.

The periodic beacon mechanism is used to pick up one-hop neighbor information, including depth, address and the pairwise distance. All nodes can get their depths using a pressure sensor [13]. The periodic beacon mechanism adopts a simple time-based schedule. Nodes broadcast the beacon message to its neighbors during the specific slot in turn. The slot length is related to the maximum propagation delay of node communication range. When neighbors receive the beacon message, they can compute the distance information by ToA (Time of Arrival) technique [25], and pick up depth and address information embedded in the beacon. The periodic beacon mechanism is scheduled with a period *T* to update the neighbor information. Two-hop neighbor information is exchanged following the beacon process with the same time-based schedule as the beacon period. Each node broadcasts its neighbor information in turn, and the others collect the two-hop neighbor information. Besides, the time-based schedule requires time synchronization for transmission scheduling; in recent years, several works [26,27,28] have provided time synchronization mechanisms for UWSNs. Hence, the time synchronization issue is out of the scope of this paper.

### 3.2. Path Diversity Improved Forwarding Mechanism

Supposing neighbor set $V_{i}=\left\{1,2,\ldots ,k,l\right\}$ belongs to node *i*, when node *k* and *l* are deployed closer to each other in geography, there exists two potential risks: (i) If node *k* fails to receive packets from node *i* when the channel quality is bad, the probability of successfully decoding the packets by node *l* could also be very small. It is unwise for node *i* to still select node *l* as a next-hop forwarder when node *k* fails to relay packets. It will lead to increase in energy consumption and latency. (ii) Although the closely-positioned receivers can both successfully decode the packets with a good quality channel, it will impose restrictions on the candidates selection at next-hop. On the one hand, it cannot provide more choice for next-hop candidates selection, since the neighbors of node *k* and *l* are almost the same. On the other hand, once node *k* is located at the local maximum point, node *l* may also have no available neighbor with a lower depth.

The intuition behind the above example is that channel quality and candidate location distribution are two important factors affecting packet transmission in multi-hop UWSNs. The packet reception depends on the channel quality. The candidate distribution affects the path connectivity, and the closely-positioned candidate nodes is not a good choice for packet forwarding. Although EPA-based candidate selection method considers the underwater fading channel, unfortunately, it ignores the candidate location distribution problem.

To provide the reliable and diverse path for packet forwarding, we propose a path correlation based normalized sum of neighbors advancements, called EEPA (Enhanced Expected Packet Advance), for candidate set selection. It is defined by Equation (4). Our object is to maximize the forwarding advancements of the candidate set under the restriction of path correlation.

(4) $$\mathrm{EEPA}\left(V_{s}\right)=\sum_{i=1}^{k}d_{si}^{z}p_{si}\prod_{j=0}^{i-1}\left\{1-p_{sj}\rho \left(s,i,j\right)\right\}$$

The neighboring set $V_{s}$ belongs to current forwarding node *s*, including *k* nodes, where $p_{si}$ is the packet successfully delivery probability over link (s,i) and $p_{s0}$ is defined as 0. $d_{si}^{z}$ represents the depth between node *s* and node *i*, given by $d_{si}^{z}=d_{s}-d_{i}$. ρ(s,i,j) represents the path correlation between neighbor nodes *i* and *j*. Since in [29] we employ Binary Phase Shift Keying (BPSK) modulation in the physical layer , the probability of bit error is given by Equation (5):

(5) $$p_{e}\left(s,i\right)=\frac{1}{2}\left(1-\sqrt{\frac{\gamma (s,i)}{1+\gamma (s,i)}}\right)$$

Thus, in [14], the packet successfully delivery probability of a packet with *m* bits size over link (s,i) is given by Equation (6):

(6) $$p_{si}={\left(1-p_{e}\left(s,i\right)\right)}^{m}$$

With EEPA metric, the packet forwarding advancements provided by node *i*, $i\in V_{s}$, consists of two parts: one is the advancements related to the distance based channel quality, and the other is the path correlation between nodes *i* and *j*, where node *j*, $j\in C_{s}$, already exists in the current candidate set. There are two practical path correlation coefficients proposed in this paper. One is represented by the normalized distance between two neighbors, named PICS, the path correlation coefficient being given by $\rho \left(s,i,j\right)=1-d_{ij}^{e}/R$, $d_{ij}^{e}<R$. To reduce the routing cost, the other path correlation coefficient is represented by a random value, ρ(s,i,j)∈(0,1), named PRCS.

The EEPA metric is defined as a trade-off between forwarding advancements and path correlation. A shorter distance $d_{ij}^{e}$ means a higher path correlation between nodes *i* and *j*, then, the factor ρ(s,i,j) will reduce the advancements carried by node *i*. When the path correlation ρ(s,i,j) is lower, the packet reception of node *i* mostly depends on the progress provided by itself. Thus, the neighbor nodes which not only can provide higher progress but also have a lower path correlation with forwarding nodes already in the candidate set will be selected as the next potential forwarder. The normalized distance based path correlation can effectively avoid forwarding node concentrate in geographical. It can provide the diverse path in current forwarding hop, and offer more choice for the forwarding nodes selection at next hop. Although the random correlation coefficient cannot accurately represent the location relationship between neighbors, the PRCS method has two advantages. One is that it does not need two-hop neighbor information, and the other is that we use a broader candidates selection range. Then, the random correlation could also diversify the forwarding paths.

It is computationally hard that finding a forwarding set with maximum EPA value [14]. It is similar to EEPA metric. In this paper, we propose two heuristics to search for a cluster that maximizes EEPA with different candidates selection range. In PICS, the path correlation coefficient is supported by two-hop neighbor information, making it so the candidate nodes should be in communication range of each other. The random path correlation coefficient in PRCS allows the candidates could be out of the communication range of each other.

**A. Candidates Selection Algorithm for PICS**: The hidden terminal problem is that nodes in the candidate set placed beyond the communication range of each other cannot notice the transmission of any packet by other candidates, which leads to redundant transmissions and packet collisions. To avoid the hidden terminal problem, the PICS algorithm requires that all candidate nodes be a neighbor of each other. As a result, all candidate members can hear each other directly. Thus, PICS should be supported by two-hop neighbor information. The candidates selection algorithm for PICS is shown in Algorithm 1. Suppose node *j* is a neighbor of node *s*, the NADV of packet forwarding from node *s* to *j* is given by $\frac{d_{sj}^{z}}{1/p_{sj}}=d_{sj}^{z}p_{sj}$. The PICS algorithm starts from the neighbor node *j* with the maximum NADV value, set as $c_{0}$ (line 1). Then, find the neighbor node *i*, $i\in V_{c_{0}}$, with the maximum EEPA value, set as $c_{1}$. Next, find the neighbor node *l*, $l\in V_{C_{s}},C_{s}=\left\{c_{0},c_{1}\right\}$ with the maximum EEPA value. Repeat the above loop until no node left within the communication range of all the node in candidate set $C_{s}$ (Lines 3–8).

We define a standard plane formed by the candidate node $c_{0}$, $c_{1}$ and $c_{2}$, the standard depth is defined as $D_{z}=\sum_{i=0}^{2}\left(d_{s}-d_{c_{i}}\right)/3$. When depth difference between the candidate node $c_{i}$, i>2 and its neighbor node *j*, $j\in V_{c_{i}}$ satisfies Equation (7)

(7) $$d_{c_{i}}-d_{j}\geq D_{z}+D_{r}$$

where $D_{r}$ is a depth threshold. Then, the packet forwarding carried by candidate node $c_{i}$ is considered as an effective forwarding; otherwise, it is an ineffective forwarding and the candidate node $c_{i}$ should be removed from the candidate set $C_{s}$ (line 10).

**Algorithm 1** Candidates Selection Algorithm-PICS
1:Start from node *j* with maximum NADV, set as $c_{t=0}$. {Node *s* is the current forwarder}2: 3:
**repeat**
4: Find node $i\in V_{C_{s}}$5: 6: Set node *i* with maximum $EEPA(V_{C_{s}})$ as next candidate node $c_{t=t+1}$7: 8:**until** No node satisfies $i\in V_{C_{s}}$9: 10:Remove node *i* from the candidate set $V_{C_{s}}$ when it does not meet Equation (7)


**B. Candidates Selection Algorithm for PRCS**: Further, the candidates selection range will restrict the location of candidate nodes. To avoid the hidden terminal problem, many sender-based candidate selection procedures require all nodes in the candidate set to be able to hear each other directly. When a node successfully forwards the packets, the others could know it and give up the current forwarding. However, it is an useful solution to reduce redundant transmissions and avoid packet collisions. It will reduce the candidate set size, causing the candidate nodes to be geographically close to each other. For example, the candidate selection algorithm proposed by Hydrocast [14] limits the candidate set size in a sphere of radius R/2, while the primary candidate set size could be a sphere of radius *R*. In addition, the EPA-based forwarding follows the link independence model, which assumes that the packet loss in a receiver has no relationship with the packet losses in other receivers [30]. Recently, the link correlation model where broadcast packet receptions among closely-positioned receivers are not independent has been considered in  [30,31,32].

To enlarge the size of the candidate set, the candidates in PRCS could be selected in the whole communication range of the current forwarder. The random path correlation coefficient allows PRCS to make routing decision only by one-hop neighbor information provided by the periodic beacon reports. The candidate set selection algorithm for PRCS is shown in Algorithm 2. We firstly rewrite the EEPA metric as $\mathrm{EEPA}\left(C_{s},i\right)=d_{i}^{z}p_{si}\prod_{j=0}^{i-1}\left\{1-p_{sj}\rho \left(s,i,j\right)\right\}$, $i,\;j\in V_{s}$, $j\in C_{s}$. Suppose node *s* is the current forwarder, we also start from the node $i,\;i\in V_{s}$ with the maximum $\mathrm{EEPA}(C_{s},i)$ value (line 2–5). The initial case is the EEPA value equal to the NADV value when i=1. Then, check whether the forwarding advancements provided by node *i* satisfies $\mathrm{EEPA}\left(C_{s},i\right)>P_{T}$ , where $P_{T}$ is the forwarding threshold. If yes, set node *i* as $c_{t}$ (Lines 7–10), otherwise, the algorithm stops (Line 12). Repeat the above loop and the specific neighbor node will be added to the candidate set in turn.

It has been proven in [18] that $EPA(V_{s})$ is a strictly increasing function of *k*. It can be inferred that $\mathrm{EEPA}(V_{s})$ also is a strictly increasing function of *k*. It means that all the neighbors of node *s* could be included in the candidate set to maximize forwarding progress. Actually, it is not wise to add all the neighbors to the candidate set, which will increase the risk of packet conflict caused by the hidden terminal. From Equation (4), the weight of $\prod_{j=0}^{i-1}\{1-p_{sj}\rho \left(s,i,j\right)$ in function $\mathrm{EEPA}(C_{s},i)$ for node *i* will decline exponentially when the number of candidate nodes increases. It causes node *i* to provide little forwarding advancements when the number of current candidate set is large. Thus, an effective forwarding threshold $P_{T}$ could prevent too many candidate nodes.

**Algorithm 2** Candidates Selection Algorithm-PRCS
1:
**repeat**
2: **for** $i\leftarrow 1,i\in V_{s}$ **do**3:  Find node *i*, with maximum $EEPA(C_{s},i)$4: 5: **end for**6: 7: **if** $EEPA\left(C_{s},i\right)\geq P_{T}$ **then**8:  Set node *i* as next candidate node $c_{t=t+1}$9: 10: **end if**11: 12:
**until** $EEPA\left(C_{s},i\right)<P_{T}$



**Figure 2:**
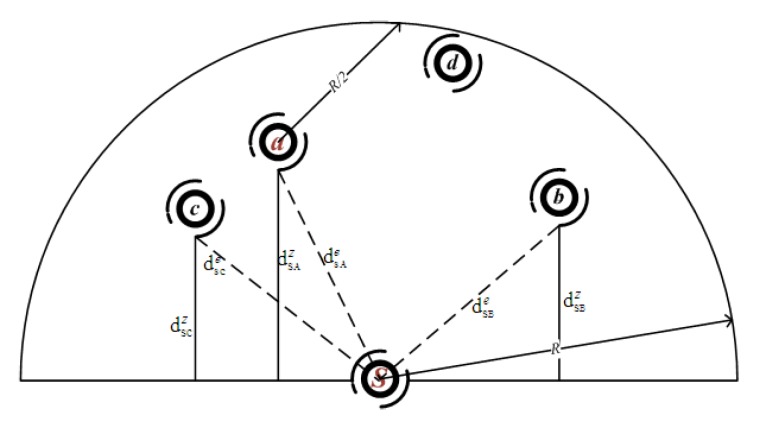
Candidates selection diagram—an example.

In Figure 2, suppose node *s* is the current forwarder and node *a* is a neighbor node with the highest NADV, only node *c* is in the R/2 communication range of node *a*. In PICS algorithm, the next candidate node will be selected in the neighbor set $V_{a}$; when $\mathrm{EEPA}\left(C_{s},d\right)>\mathrm{EEPA}\left(C_{s},c\right)$, $a,d\in V_{a}$, node *d* will be selected as a next candidate node. In PRCS algorithm, when $d_{sb}^{z}p_{sb}\left(1-p_{sa}\rho \left(s,a,b\right)\right)>d_{sc}^{z}p_{sc}\left(1-p_{sa}\rho \left(s,a,c\right)\right)$, node *b* should be selected as a next candidates member. Then, whether node *c* or *d* could be a candidate member depends on the depth, successful packet delivery probability and the path correlation with the existing candidate forwarding set $C_{s}=\left\{a,b\right\}$.

**Figure 3:**
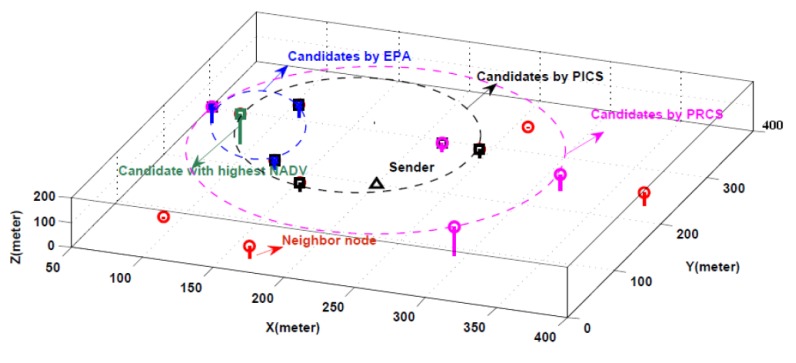
An example of one-hop candidates selection.

An example of one-hop candidate set selection is illustrated in Figure 3. The black triangle is the sender node, the red ring represents its neighbor node, and the green square shows the node with highest NADV value. Obviously, the candidate nodes selected by EPA metric are limited in a specific space, represented by blue-x. The candidate nodes chosen by PICS algorithm are scattered within the communication range of each other, represented by the black square. The candidate nodes selected by PRCS algorithm could get a larger coverage area, represented by the pink ring.

In the sender-based candidates selection scheme, the candidate priority is always based on NADV [14,32]. A dynamic priority is applied in this paper. We take the sequence that neighbor node adds to the candidate set as its priority λ. When candidates with higher priority fail to receive packets, the candidate member that has a higher path correlation with the previous forwarders will often fail to forward. The next forwarder which not only has a higher NADV value but also has a lower path correlation with the previous forwarders will have a higher priority. Node priority information is also embedded in the packet header.

### 3.3. Candidate Coordination

The time-based candidate coordination is applied in this paper. The candidate nodes with a higher priority will forward the packet earlier after its holding time expired. The others in the candidate set will only store the packet, and listen to the channel. Once they realize the packet has been forwarded, they will drop the packet; otherwise, another forwarder will be selected based on the priority until no node is left in the candidate set. The above process will be repeated at each hop until the packets arrive at the sink nodes. A linear holding time function as $f\left(\lambda_{i}\right)=\lambda_{i}\alpha$ is applied both in PICS and PRCS, where $\lambda_{i}$ is the priority and α is dynamic parameters at each hop.

**Figure 4:**
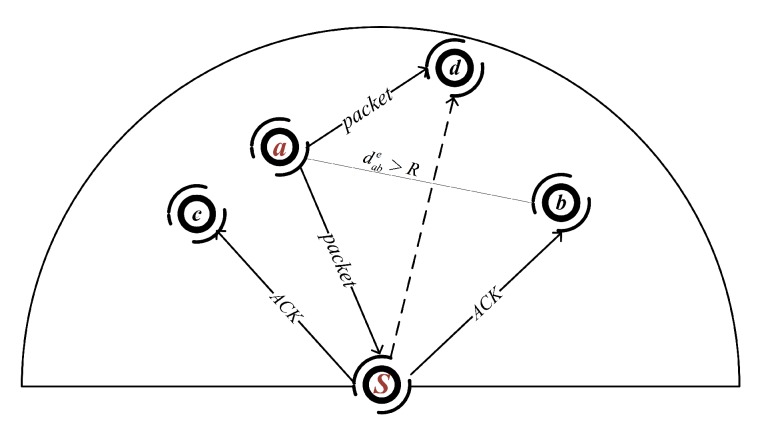
Candidate coordination diagram.

**A. Candidates coordination for PICS**: In PICS, since each candidate node can hear each other directly, as shown in Figure 4, node *a* has a higher priority than others, thus it forwards the packets earlier than others, e.g., node *d*. Then, node *d* cannot broadcast the packet before the signal from node *a* arriving. Thus, we have

(8) $$t_{s}+\tau_{sa}+\Delta +f\left(\lambda_{a}\right)+\tau_{ad}<t_{s}+\tau_{sd}+\Delta +f\left(\lambda_{d}\right)$$

where $t_{s}$ is the transmission start time of the last forwarder *s* and Δ is the packet transmission time. From Equation (8), we can get α should satisfy

(9) $$\alpha >\frac{\tau_{sa}+\tau_{ad}-\tau_{sd}}{\lambda_{d}-\lambda_{a}}$$

**B. Candidates coordination for PRCS**: The candidates selected in PRCS are allowed to be located outside the communication range of each other. We apply a skillful coordination method to handle the hidden terminal problem. As we know, all members of the candidate set are the neighbors of forwarder *s*, $C_{s}\subset V_{s}$. Forwarder *s* can be used to relay the ACK messages for suppressing redundant transmissions.

As shown in Figure 4, suppose $C_{s}=\left\{a,b,c\right\}$, node *a* has a higher priority than node *b* and *c*, while node *b* is beyond the communication range of node *a*. Suppose node *a* can successfully receive the packets from node *s* and its NADV value is more than the forwarding progress threshold, then node *a* is allowed to forward the packet immediately. When node *a* forwards the packets to its next-hop candidates, the packets will also arrive at node *s*. If node *s* hears the packets, it will broadcast an ACK message to inform the other candidates to not forward the same packets again. Once the candidate nodes receive the ACK message, they will give up the current forwarding, Otherwise, they will turn to forward the packets after the holding time expired. Since node moving or the bad communication channel quality, the worst case is that the previous forwarder *s* fails to receive the packets transmitted by the current forwarder. It will lead to redundant transmission. Similar to the traditional approach, the candidates will also give up forwarding when they hear the packets transmitted by the forwarders in the current candidate set.

The packet receiving time at node *a* is $t_{s}+\tau_{sa}+\Delta$. Then, node *a* will forward the packet at time $t_{a}=t_{s}+\tau_{sa}+\Delta +f\left(\lambda_{a}\right)$. Suppose node *s* will broadcast the ACK message once it receives the packet from node *a* with no delay; to suppress redundant transmissions, node *b* cannot broadcast the packet before receiving the ACK message, thus we can get

(10) $$t_{a}+\tau_{as}+\Delta +\tau_{sb}+\Delta_{ack}\leq t_{s}+\tau_{sb}+\Delta +f\left(\lambda_{b}\right)$$

From Equation (10), α should satisfy

(11) $$\alpha >\frac{2\tau_{sa}+\Delta +\Delta_{ack}}{\lambda_{b}-\lambda_{a}}$$

For a given candidate set, the α can be determined by examining every candidate nodes pair using two-hop neighbor information for PICS and α can also be determined by one-hop neighbor information for PRCS.

## 4. Simulation Result

We used the underwater network simulator proposed in [33]. In the simulator, each sensor node has a single, half-duplex transducer. For acoustic communications, the channel model is in Section 3.1, and BPSK modulation is implemented in the physical layer. The transmit power is 105 dB re μPa for data transmission, and 125 dB re μPa for beacon process. Ambient noise PSD is 46 dB, SNR threshold is 20 dB and the carrier frequency f=25 kHz. The packet length is 50 bits, the bit rate is 10 kbps and the acoustic speed is c=1500 m/s. A CSMA-based broadcast protocol is employed in the MAC layer. When a node has data to send, it first senses the channel. If the channel is free, then the node broadcasts the packet. Otherwise, it backs off. The maximum number of backoffs is four for one data packet [11].

The network performances were evaluated by the random deployed network. The network size was 400m×400m×800m. There was one source located at (200,200,800), and three sinks located at (100,100,10), (200,200,10) and (300,300,10). All the simulation scenarios were tested for various numbers of nodes from 100 to 350. The source node sent one packet per 10 s. For each setting, the results wer averaged over 50 runs with a randomly generated topology. The total simulation time for each run was 500 s. In all simulations, we set the parameters similar to UWM1000 [11,34]: the transmission range was 200 m, and the energy consumption on sending mode, receiving mode and idle mode was 2 W, 0.75 W and 8 mW, respectively. Note that only one sink node located at (200,200,10) was used for OVAR protocol in the simulation.

Five routing protocols were compared in the simulation: EPA is the EPA-based candidates selection algorithm proposed in Hydrocast [14]. DBR is the depth based protocol proposed in [13]. OVAR is proposed in [16]. PRCS and PICS are two protocols proposed in this paper. The network performances were measured in terms of packets delivery ratio, average energy consumption and end-to-end delay in the two scenarios static random network and random moving network. The packets delivery ratio means the number of packets successfully received compared to the number of packets that have been sent out in the network. Average energy consumption means the average energy consumed by one node for one packet. The average end to end delay refers to the average time taken for all packets to be transmitted across the network from source to sink.

**Figure 5:**
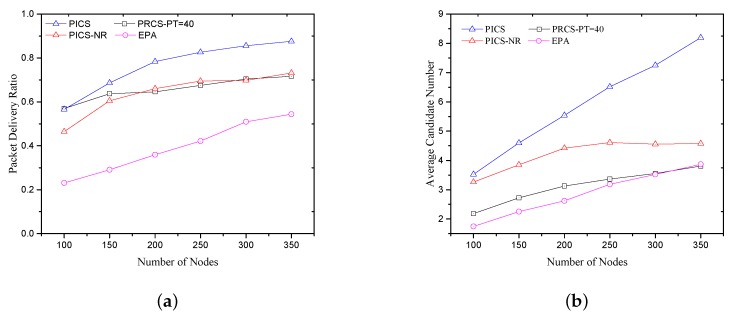
Verifying the impact of candidate set size and path correlation on network performance. (**a**) Packet delivery ratio; (**b**) Average candidate number.

**A. Verifying the impact of candidate set size and path correlation on network performance**: The simulation study below verifies the impact of candidate set size and path correlation factor on the packet delivery ratio. Figure 5a shows the packet delivery ratio (PDR) and Figure 5b gives the average candidate number at each hop. With the same channel quality, candidate location destitution significantly affects the packets forwarding. (i) In PICS, we compare the EEPA metric with path correlation factor (“PICS” in Figure 5) and without path correlation factor (“PICS-NR” in Figure 5). With the normalized distance based path correlation factor, PICS can effectively expand the range of candidate sets, resulting in a larger candidate number to improve the PDR performance. (ii) Although PRCS obtains almost the same candidate number as the EPA when the forwarding threshold is set as $P_{T}=40$ (the candidates number of PRCS is a litter higher than EPA when the network nodes number is less than 300), the PDR performance of PRCS is much higher than the EPA. It is because the larger candidate set size provided by PRCS improves the path diversity to deal with the routing void problem. (iii) Further, PRCS can still achieve almost the same PDR performance as the PICS without path correlation factor (PICS-NR), while its average candidate number is smaller than that one. That is also related to candidate set size. Based on the above simulation results, we can conclude that the path correlation factor can enlarge the candidate set size to allow more nodes to participate in forwarding, and a larger candidate set size can supply the diverse path for forwarding to improve the PDR performance.

**B. Compared with the previous solutions in static scenario**: Except for the source and destination nodes, other nodes are randomly deployed in the network and do not move. In the simulation, we set the depth threshold parameter as $D_{r}=20$ m in PICS, and the depth threshold is 60 m in DBR. Various forwarding thresholds, $P_{T}=\left\{20,30,40\right\}$, are examined in PRCS.

**Figure 6:**
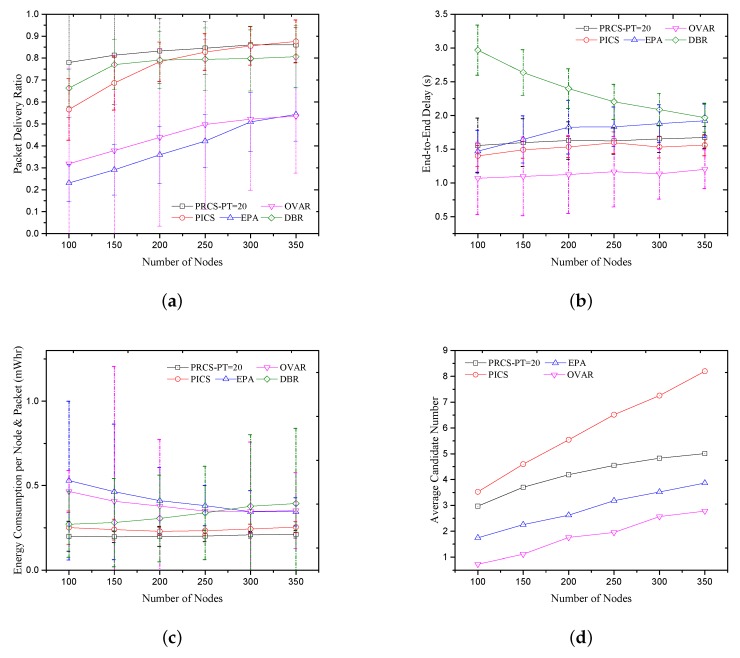
Compared with the previous solutions in static scenario. (**a**) Packet delivery ratio; (**b**) End-to-End delay; (**c**) Energy consumption per node and packet; (**d**) Average candidate number.

With the increased number of candidate nodes, as shown in Figure 6d, the PDR performance of EPA, DBR, OVAR, PICS and PRCS increase, as shown in Figure 6a. Recall that DBR applies a greedy forwarding scheme with an opportunistic forwarding flavor. It will deliver packets in multiple paths to improve reliability. This makes DBR achieve a better PDR performance than EPA and OVAR, especially when the number of nodes is small (the PDR performance is better than PICS when the number of nodes is less than 200). Due to the conflict among multiple paths, the PDR performance cannot improve well with the increase in the number of nodes. Both PRCS and PICS obtain higher PDR performance than others mainly for two reasons. One is that the candidate number of PICS and PRCS is greater than others, as shown in Figure 6d, which is caused by the larger candidate set sizes. The other is that, with EEPA metric, the larger candidate set sizes could output an available candidates location destitution to mitigate routing void risk. Moreover, as shown in Figure 6d, PRCS employs fewer candidate nodes than PICS for forwarding, but the PDR performance of PRCS is always higher than PICS when the number of nodes is less than 300. It also benefits from the larger candidate set sizes. The increased number of candidates node and the more local topology information cause PDR performance of PICS is better higher than PRCS when the number of nodes is 350. Although the conflicts in the beacon process cause the average candidate number of OVAR to be fewer than EPA, as shown in Figure 6d, the PDR performance of OVAR is still higher than EPA for two reasons. One is OVAR adopts a larger candidate set size than EPA, and the other is the beacon process in OVAR established the path connectivity from the source node to the sink.

Figure 6b measures the average end-to-end delay of the five protocols. The holding time in DBR is $t_{dbr}\left(d\right)=\frac{2\tau }{\delta }\left(R-d\right)$, where τ is the maximal propagation delay of one-hop, *d* is the depth difference between the current node and the previous forwarder. We set δ=R/4 in the simulation. In EPA, the holding time is given as f(d)=α(R−d) and α is the dynamic parameters in each hop, which we set as α<0.01 in the simulation. OVAR gets the lowest end-to-end latency because it can capture the minimum number of hops on the routing path from the source to the sink, and fewer candidate nodes also reduce the total holding time per hop. The higher end-to-end delay result in DBR is mainly caused by many redundant transmissions. PRCS and PICS achieve a lower end-to-end delay than DBR and EPA. The reason is that, when the candidate node with higher priority abandons forwarding due to bad channel quality or the candidate node is located at a local maximum point, PRCS and PICS will choose the next forwarder that not only has higher forwarding advancement but also a lower path correlation with the previous one. It can help the forwarding escape from the routing void zone quickly. In PICS, the forwarding depth threshold can also avoid partially invalid forwarding and ensure the packets can be forwarded to a neighbor with a lower depth than the average depth of the current candidate set. Because of the two-way message interaction, the delay of PRCS is greater than PICS.

We plot the average energy consumption of the five protocols in Figure 6c. Energy consumption includes two parts in the simulations, packets transmission and neighbor information exchanging. PRCS only needs one-hop neighbor information supported by the beacon period, while two-hop neighbor information is exchanged in PICS, EPA and OVAR. Although all routing decisions in DBR are made locally, DBR results in higher average energy consumption due to multi-path forwarding and packet collision. The higher PDR and effective retransmission suppression make PICS and PRCS have a lower average energy consumption than others.

**Figure 7:**
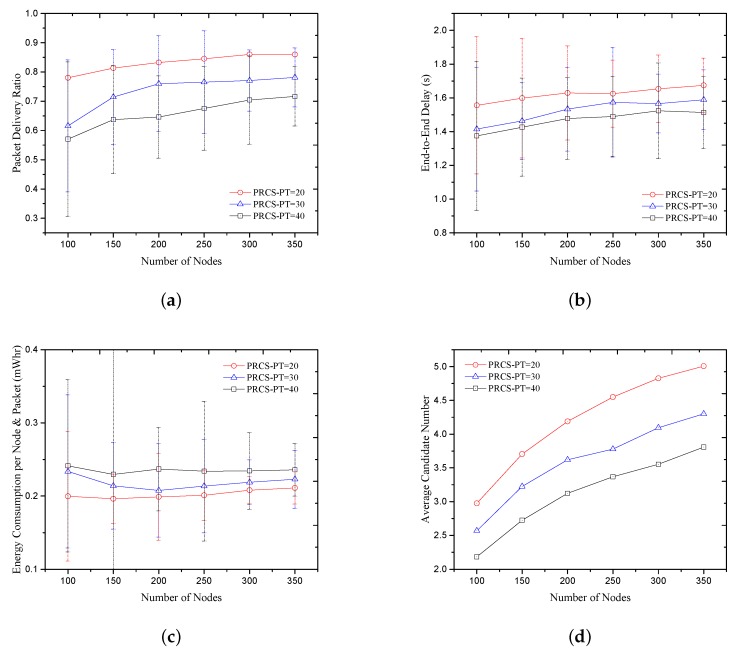
PRCS with different forwarding progress threshold. (**a**) Packet delivery ratio; (**b**) End-to-End delay; (**c**) Energy consumption per node and packet; (**d**) Average candidate number.

We compare the PRCS protocol performances with different forwarding threshold in Figure 7. With the decrease of forwarding threshold $P_{T}$, the average candidate number increases, as shown in Figure 7d. It makes the PDR of PRCS protocol increase, as shown in Figure 7a. In addition, it causes the end-to-end delay to increase and the average energy consumption to decrease.

**Figure 8:**
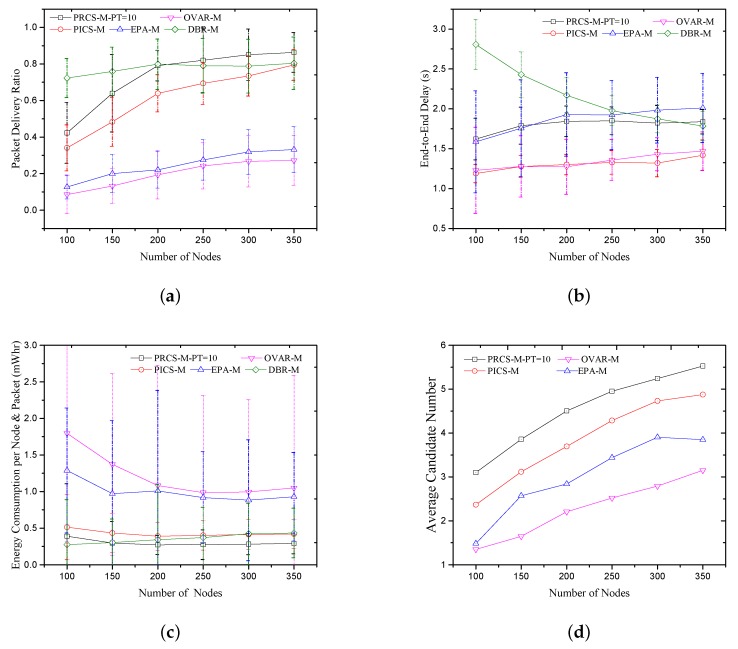
Compared with the previous solutions in mobile scenario. (**a**) Packet delivery ratio; (**b**) End-to-End delay; (**c**) Energy consumption per node and packet; (**d**) Average candidate number.

**C. Compared with the previous solutions in mobile scenario**: All nodes move except for the source and sink node and follow the model in [11]. We also measured the performance of PDR, average energy consumption and end to end delay, as shown in Figure 8. The minimum speed is 0 m/s and the maximum speed is 1 m/s. In the simulation, we set the depth threshold parameter as $D_{r}=20$ m in PICS, the holding time parameter meets α<0.02 in EPA, the forwarding threshold is $P_{T}=10$ in PRCS, and the neighbor information update period is T=150 s. To reduce the effect of nodes moving on the accurate neighbor information, we set a dynamic slot length that is related to half of the maximum propagation delay of node communication range multiply by a random value in the beacon process.

In Figure 8a, the PDR performances of the five protocols in the mobile scenario are plotted. The PDR performance of DBR does not vary much with node moving, since all routing decisions in DBR are made locally based on a node’s depth information. No topology or route information needs to be exchanged between neighboring nodes. It makes DBR have a better PDR performance, especially in the sparse network (e.g., nodes number less than 200). The PDR of OVAR, EPA, PICS and PRCS protocol decline significantly in mobile scenarios caused by the outdated neighbor information and routing information. As shown in Figure 8d, the candidate number of PICS declines seriously, but PRCS could provide enough candidate nodes with the appropriate forwarding threshold. Compared with EPA and OVAR, the larger candidate set size employed by PRCS and PICS will bring more candidate nodes involved in forwarding, which is conducive to node movement. In addition, the PDR performance of OVAR is lower than others, mainly because the available routing information is more likely to fail over time when there are fewer candidate nodes.

As shown in Figure 8b, in PICS, the movement of nodes will decrease the number of candidate nodes, resulting in a smaller holding time at each hop. Thus, the end-to-end delay of PICS declines relative to the static scenario. Because of the neighbor information updating, the average energy consumption of OVAR, EPA, PICS and PRCS increases, as shown in Figure 8c, especially in OVAR and EPA that require two-hop neighbor information. With the increase of energy consumption and the decrease of the PDR, the average energy consumption of PICS is worse than DBR when the network number of nodes is less than 300. Except the case that the network node number is 100, the PRCS protocol always consumes the minimum average energy because it only requires one-hop neighbor information and achieves good PDR performance.

**Figure 9:**
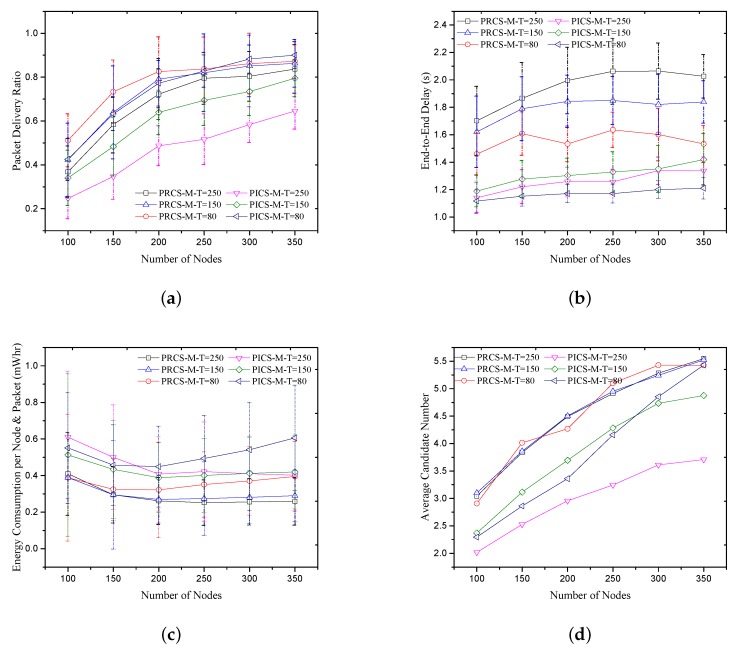
PRCS/PICS with different neighbor information update interval. (**a**) Packet delivery ratio; (**b**) End-to-End delay; (**c**) Energy consumption per node and packet; (**d**) Average candidate number.

**D. PRCS/PICS with different neighbor information update interval**: We compared the PRCS and PICS protocols performances with varied beacon update interval T={80,150,250}. The simulation results of PDR, average energy consumption and end-to-end delay are presented in Figure 9. By increasing the beacon update interval, the PDR performances of both PRCS and PICS are decreased because the neighbor information is fading over time. The outdated information leads to increased latency in PRCS protocol. In PICS, when the beacon update interval is T=250, the average candidate number is fewer than for T=150, as shown in Figure 9d. It leads to a smaller holding time at each hop. Then, its end-to-end delay is lower than for T=150. With decreasing of the beacon update interval, the energy consumption by neighbor information exchanging will increase, which makes both PICS and PRCS cost more energy when T=80, especially with more nodes in the network (e.g., more than 200 nodes in PICS and more than 150 nodes in PRCS).

## 5. Discussion and Conclusions

The existing research on opportunistic routing mainly ignores three issues: (i) GPS information is inapplicable in marine applications; (ii) candidate selection algorithms only consider the forwarding advancements at the current hop, ignoring the influence of the position distribution of candidate nodes; and (iii) to mitigate the hidden terminal problem, the candidate set size is restricted. To solve the above problems and design green routing protocol, in this paper, we propose a path diversity improved strategy in candidate selection procedures. It can provide diverse paths for packets forwarding by taking the distribution of candidate nodes into account, mitigating the impact of routing void problem and improving packet forwarding efficiency. Based on the EEPA metric, we propose two effective location-free routing protocols, PICS and PRCS, for UWSNs. PICS uses two-hop neighbor information to make routing decisions and PRCS only needs one-hop neighbor information. Both PRCS and PICS protocols have good performance; in particular, PRCS protocol has better performance in terms of PDR and average energy consumption, while PICS protocol achieves lower end-to-end delay performance and better PDR performance when the number of network nodes is large.

Based on the simulation above, we find the following. (i) The candidate location distribution and candidate set size are important to improve the packet delivery ratio. A larger candidate set size could bring more nodes and an effective candidate location distribution could supply diverse paths for forwarding. (ii) More candidate nodes will increase the end to end delay. On the contrary, fewer candidate nodes cannot always satisfy the packets delivery ratio. It is worth considering how to obtain a higher packets delivery ratio and lower end to end delay with fewer candidate nodes. Overall, the PRCS protocol presented in this paper is an effective solution. (iii) Neighbor discovery and information updating seriously affect network performance in sender-based OR protocols, especially in energy consumption. Our future work will focus on duty-cycled based opportunistic routing protocols to improve energy efficiency.
